# Preventive Effects of Tocotrienol on Stress-Induced Gastric Mucosal Lesions and Its Relation to Oxidative and Inflammatory Biomarkers

**DOI:** 10.1371/journal.pone.0139348

**Published:** 2015-10-14

**Authors:** Mohd Fahami Nur Azlina, Yusof Kamisah, Kien Hui Chua, Ibrahim Abdel Aziz Ibrahim, Hj Mohd Saad Qodriyah

**Affiliations:** 1 Department of Pharmacology, Faculty of Medicine, Universiti Kebangsaan Malaysia, Kuala Lumpur, Malaysia; 2 Department of Physiology, Faculty of Medicine, Universiti Kebangsaan Malaysia, Kuala Lumpur, Malaysia; 3 College of Medicine, Umm Al Qura University, Makkah, Saudi Arabia; Center for Cancer Research, National Cancer Institute, UNITED STATES

## Abstract

This study aimed to investigate the possible gastroprotective effect of tocotrienol against water-immersion restraint stress (WIRS) induced gastric ulcers in rats by measuring its effect on gastric mucosal nitric oxide (NO), oxidative stress, and inflammatory biomarkers. Twenty-eight male Wistar rats were randomly assigned to four groups of seven rats. The two control groups were administered vitamin-free palm oil (vehicle) and the two treatment groups were given omeprazole (20 mg/kg) or tocotrienol (60 mg/kg) orally. After 28 days, rats from one control group and both treated groups were subjected to WIRS for 3.5 hours once. Malondialdehyde (MDA), NO content, and superoxide dismutase (SOD) activity were assayed in gastric tissue homogenates. Gastric tissue SOD, iNOS, TNF-α and IL1-β expression were measured. WIRS increased the gastric MDA, NO, and pro-inflammatory cytokines levels significantly when compared to the non-stressed control group. Administration of tocotrienol and omeprazole displayed significant protection against gastric ulcers induced by exposure to WIRS by correction of both ulcer score and MDA content. Tissue content of TNF-α and SOD activity were markedly reduced by the treatment with tocotrienol but not omeprazole. Tocotrienol significantly corrected nitrite to near normal levels and attenuated iNOS gene expression, which was upregulated in this ulcer model. In conclusion, oral supplementation with tocotrienol provides a gastroprotective effect in WIRS-induced ulcers. Gastroprotection is mediated through 1) free radical scavenging activity, 2) the increase in gastric mucosal antioxidant enzyme activity, 3) normalisation of gastric mucosal NO through reduction of iNOS expression, and 4) attenuation of inflammatory cytokines. In comparison to omeprazole, it exerts similar effectiveness but has a more diverse mechanism of protection, particularly through its effect on NO, SOD activity, and TNF-α.

## Introduction

Stress is well known to be associated with the formation of gastric ulcers. The ulcers frequently emerge as a result of major stressful events including surgery, trauma, shock, sepsis, and burns. The pathological basis for the development of these ulcers are reported to be multifactorial, a combination of increased gastric acid secretion[1], oxidative damages [2–4], reduction of gastric mucosal blood flow [5], inhibition of gastric mucosal prostaglandin synthesis[6,7], and inflammatory responses[8].

Nitric oxide (NO) is a biologically active substance, that is produced from L-arginine via a Ca^2+^ dependent constitutive NO synthase (cNOS) or a Ca^2+^-independent inducible NO synthase (iNOS). Nitric oxide is a potent vasorelaxant involved in the control of the gastric blood flow and is a gaseous mediator contributing to the maintenance of gastric mucosal integrity [9]. Inhibition of NOS causes a decrease in local NO production, impairs gastric microcirculation, and aggravates gastric lesions induced by noxious agents [10]. Although this is true, excessive NO production can create free radicals that have a negative effect on the gastric microenvironment. Kwiecien et al. [9] demonstrated that water-immersion restraint stress (WIRS) provokes acute inflammatory responses with interleukin-1β (IL-1β) and tumor necrosis factor-α (TNF)-α acting as the major pro-inflammatory cytokines mediated by neutrophil infiltration in gastric mucosa. Neutrophils produce superoxide radical anions (O_2_^•-^), which react with cellular membrane lipids, leading to the formation of lipid peroxides that are metabolised to end products like malondialdehyde (MDA) and 4-hydroxynonenal (4-HNE) [11].

The gastric endogenous antioxidant system works in tandem to fight oxidative damage [12]. Superoxide dismutase (SOD), an antioxidant enzyme, is the first line of defence, that catalyses the dismutation of superoxide anions into less noxious hydrogen peroxide (H_2_O_2_), which is further inactivated by glutathione peroxidase [13]. Hence, a compound with pure antioxidant properties may be potentially useful in minimising gastric mucosal damage caused by free radicals.

Vitamin E both tocopherol and tocotrienol [7,14] have been demonstrated to prevent gastric mucosal development in rats exposed to stress. Both possess antioxidant properties, but tocotrienol was reported to possess a better antioxidant capability compared to tocopherol [15]. The diverse etiological factors underlying gastric ulcers formation and the complex nature of pathways participating in healing always make gastric ulcers treatment and prevention a complicated challenge [16]. Tocotrienol had been shown to not only possess antioxidant property but also anti-inflammatory effects [17], which could be beneficial to reduce stress-induced gastric damages.

Water-immersion restraint stress (WIRS) has been used as an experimental model in the evaluation of antiulcer activity due to its reproducibility [14,18]. This model mimics clinical acute gastric lesion formation as a consequence of major trauma, surgery, or sepsis, being widely accepted for studying the mechanism of stress-induced gastric lesions [9,11]. WIRS allows for the evaluation of the stress ulcer inhibiting effects of drugs and plant-derived antiulcer preparations.

Tocotrienol extracted from palm oil which contained approximately 90% tocotrienol was used in this study. The present study evaluated the limited information on the gastroprotective activity of tocotrienol with relation to NO, pro-inflammatory cytokines and lipid peroxidation. Possible mechanisms of action are elucidated using rodents in experimental model of stress with the purpose of contributing to a better understanding of the physiopathology of gastric injury. In this study, tocotrienol was compared with omeprazole, one of the commonly prescribed drugs for the treatment of peptic-ulcer disease in a clinical setting.

## Material and Methods

Male *Wistar* rats (n = 28) were divided into four equally sized groups. Two control groups were fed with normal rat diet (NS and S) while the treatment groups received the same diet but with oral supplement of tocotrienol (TT) or omeprazole (OMZ) at 60 mg/kg and 20 mg/kg body weight respectively for 28 days. The tocotrienol dose was chosen was based on our previous studies which had shown a protective effect of tocotrienol on stress-induced gastric lesions [19,20]. Tocotrienol and omeprazole were diluted in vitamin-free palm oil which acted as a vehicle and administered by oral gavage using an 18G gavage needle. Both control groups were sham-administered with vitamin-free palm oil. At the end of the treatment period, the rats from one control group (stressed control) and both of the treated groups were exposed to water-immersion restraint stress, by placing them in individual plastic restrainer measuring approximately 17 x 5-cm and immersing them in water neck deep for 3.5 hours once, as previously described by Ibrahim et al. [14]. Following the restraining procedure the rats were sacrificed by exsanguination under anesthesia. The stomachs were then dissected along the greater curvature. The dissected stomachs were taken for evaluation of gastric ulcers, gastric malondialdehyde (MDA) content, superoxide dismutase (SOD) activity and mRNA expression, nitric oxide (NO) content and mRNA expression, interleukin 1-beta (IL-1β) and tumor necrosis factor alpha (TNF-α) protein expression. Gastric tissues were homogenized using Omni Bead Ruptor machine at 25°C with the speed of 8 m/s for 20 seconds. Homogenates produced were centrifuge at 1500 x g for 5 minutes at 4°C. The supernatant were taken for parameter measurements.

All rats were kept on a regular night/day cycle, with natural light for a period of 12 hours (0700 to 1900 h). Throughout the feeding period all rats were habituated to handling to reduce their stress-related disturbances. The rats were housed in large cages with wide wire-mesh bottoms to prevent coprophagy. Food and water were given *ad libitum* throughout the experiment. Ethical approval was obtained from University Kebangsaan Malaysia Animal Ethics Committee (UKMAEC). Humane methods of euthanasia was practiced which was exsanguination under anaesthesia following guidelines and approval from UKMAEC. The anaesthetic agents used was a combination of ketamine and xylazine at 1:1 ratio.

### Assessment of Stress-Induced Gastric Lesions

The microscopic assessment of stress-induced gastric lesions in the gastric mucosa was performed by two independent examiners who were blinded to the treatment. The assessment of lesions was done according to a quantitative scale. Lesion size in millimeter was determined by measuring each lesion along its greatest diameter. Five petechiae lesion is equal to 1 mm lesion. The total lengths in each group of rats were averaged and expressed as the lesion index; this method was modified from the previously described by Ibrahim et al. [14].

### Measurement of gastric Malondialdehyde content & superoxide dismutase activities

The content of malondialdehyde (MDA) in the stomach was determined using the method described by Ledwozyz et al. [21] with modification described by Kamisah et al. [22]. The malondialdehyde content was expressed as mmol/g tissue. Measurement of superoxide dismutase activities was done using superoxide dismutase assay kit (Item No. 706002). The kit was acquired from Cayman Chemical Company, USA.

### Measurement of tissue Nitric Oxide (NO)

Tissue Nitric Oxide was measured according to the manufacturer instruction using the QuantiChrom^TM^ Nitric Oxide Assay Kit (D2NO-100). This BioAssay Systems' Assay Kit was designed to accurately measure NO production following reduction of nitrate to nitrite using improved Griess method. The absorbance was read at 540 nm.

### Superoxide dismutase & Inducible Nitric oxide mRNA quantitation

The superoxide dismutase and iNOS mRNA quantitation were done using the standard QuantiGene Plex 2.0 assay kit (Genospectra, Fremont, CA) protocol. Briefly, the tissue lysate was transferred to a capture well in the presence of the gene-specific probe set and then hybridized at 53°C overnight. Wells were washed twice with bDNA wash buffer and then incubated at 46°C sequentially with an amplifier and an alkaline phosphatase-linked label probe with a wash step between the incubations. After the final wash step, the addition of streptavidin phycoerythrin (SAPE) generated a signal that was proportional to the amount of target RNA present in the sample. The luminescence signal was detected using a Luminex instrument. The protocol followed was as previously described by Zhang et al. [23].

### Assay of TGF-α and IL-1β protein expression

Gastric tissue TGF-α and IL-1β was assayed according to the manufacturers’ instructions using the Procarta cytokine assay kit from Panomics (Affymetrix, USA) and analyzed using a Luminex 200 (Luminex Corporation). Procarta protein assays use the xMAP® technology (mutli-analyte profiling beads) to enable quantitation of multiple protein targets simultaneously. The xMAP system combines a flow cytometer, fluorescent-dyed microspheres (beads), lasers and digital signal processing to effectively allow multiplexing of up to 100 unique assays within a single sample.

### Statistical Analysis

Statistical analysis was carried out using PRISM software version 6.00 (Graphpad, San Diego, CA). The results are expressed as mean ± standard error of mean. Statistical significance (P<0.05) was determined by ANOVA and Tukey’s post-hoc test.

## Results

### Gastric Lesions

**Fig 1:**
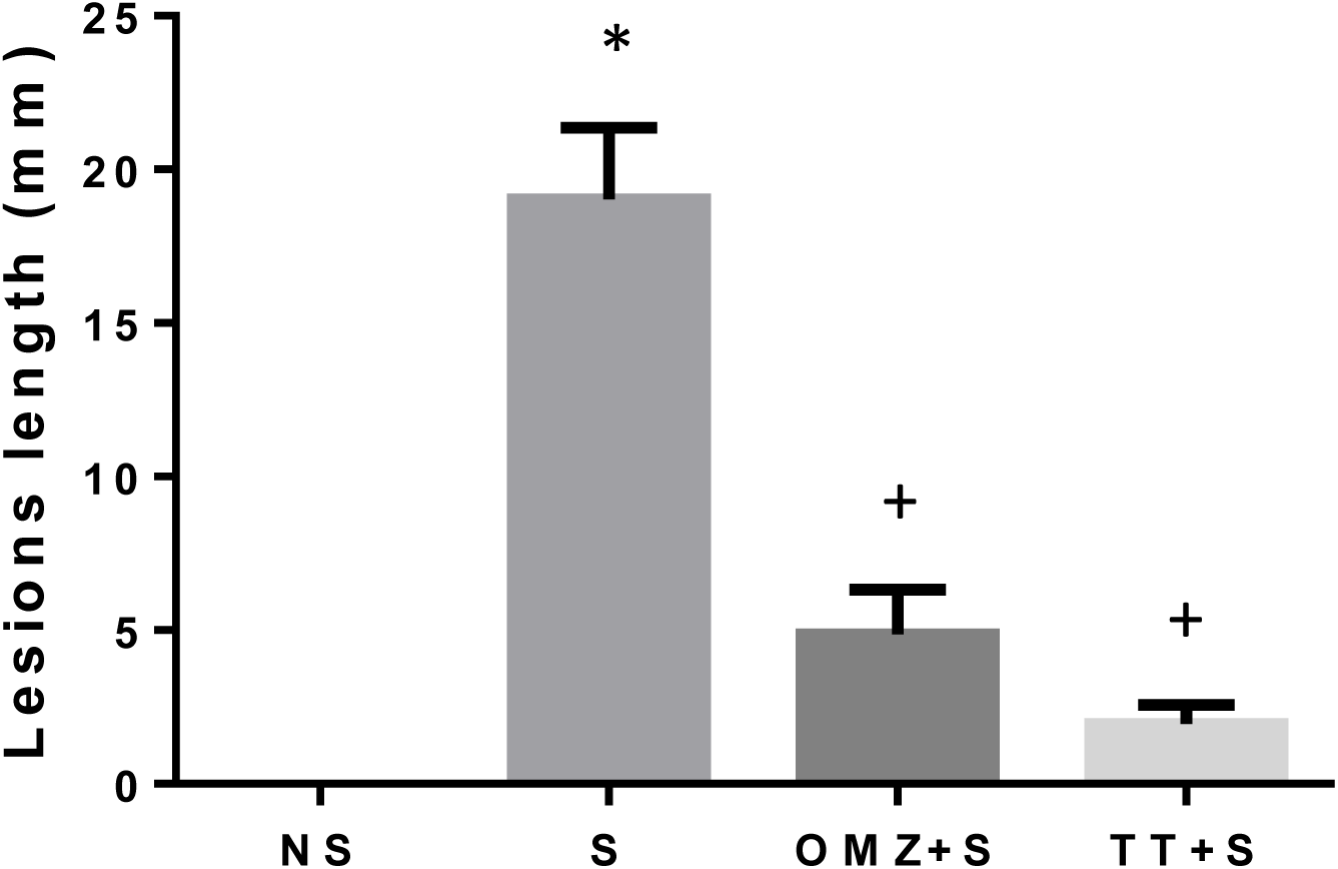
Effects of pre-treatment with tocotrienol (60 mg/kg BW) (TT) or omeprazole (20 mg/kg BW) (OMZ) on gastric lesions formation in rats exposed to water immersion restraint stress (n = 7). * vs non-stressed control (NS) (P < 0.05). ^+^vs stressed control (S) (P < 0.05).

Rats exposed to WIRS for 3.5 hours showed presence of considerable ulcers in the form of gastric erosion and haemorrhagic mucosal lesions confined to the corpus (glandular part of the stomach). As shown in Fig 1, the gastric lesion index (length in mm) in the stressed control (S) group was higher by 84.6% (P = 0.0001) compared to the tocotrienol treated group and 74.4% (P = 0.0001) compared to the omeprazole treated group. These findings indicate that pre-treatment with 60 mg/kg tocotrienol (TT) and 20 mg/kg omeprazole (OMZ) provides protection against stress-induced gastric lesions. Intact gastric mucosa did not exhibit any macroscopic lesions.

### Gastric Malondialdehyde, SOD activity, and expression

**Fig 2:**
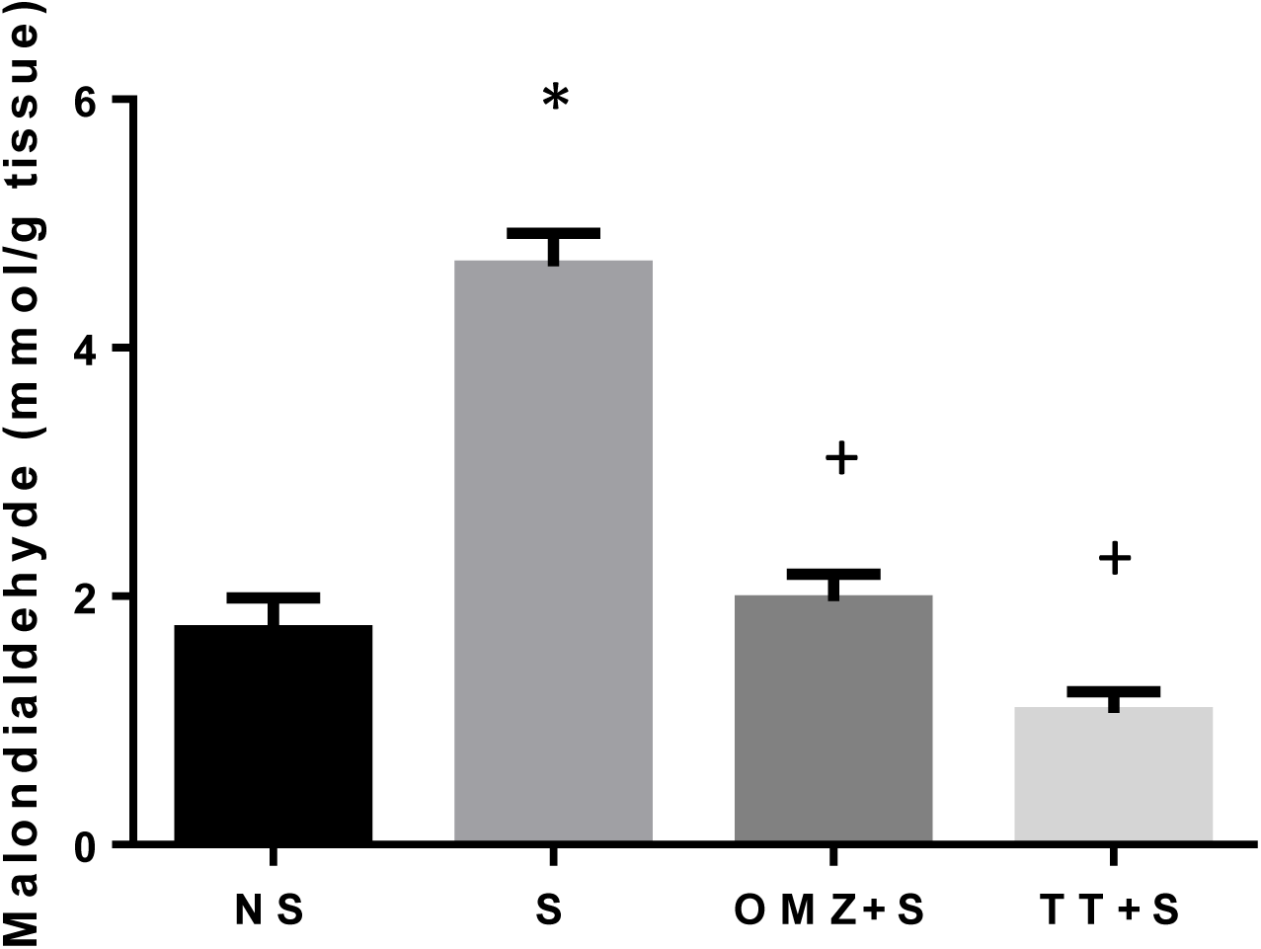
Effects of pre-treatment with tocotrienol (60 mg/kg BW) (TT) or omeprazole (20 mg/kg BW) (OMZ) on gastric malondialdehyde concentration in rats exposed to water immersion restraint stress (n = 7). * vs non-stressed control (NS) (P < 0.05). ^+^ vs stressed control (S) (P < 0.05).

Malondialdehyde (MDA) content in intact mucosa was considerably low, as shown in Fig 2. After 3.5 hours of WIRS, the MDA content increased almost threefold (P<0.0001). Pre-treatment with tocotrienol and omeprazole prevented the increase of MDA content to a level not significantly different from the recorded non-stress control. No significant difference was observed when comparing the lipid peroxidation between the TT and the OMZ stressed groups.

**Fig 3:**
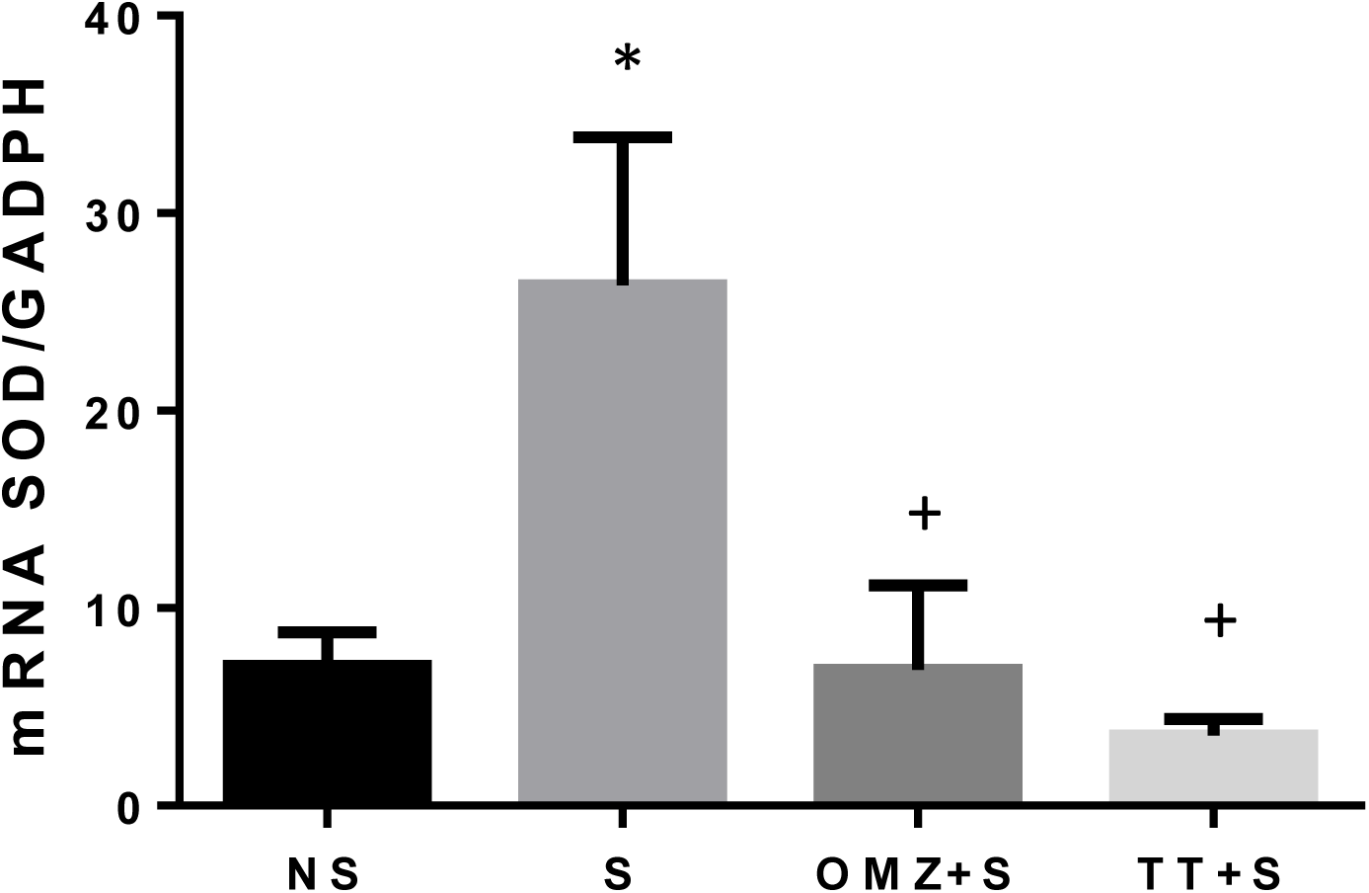
Effects of tocotrienol (60 mg/kg BW) (TT) or omeprazole (20 mg/kg BW) (OMZ) on gastric SOD mRNA expression in rats exposed to water immersion restraint stress (n = 7). * vs non-stressed control (NS) (P < 0.05). ^+^ vs stressed control (S) (P < 0.05).

**Fig 4:**
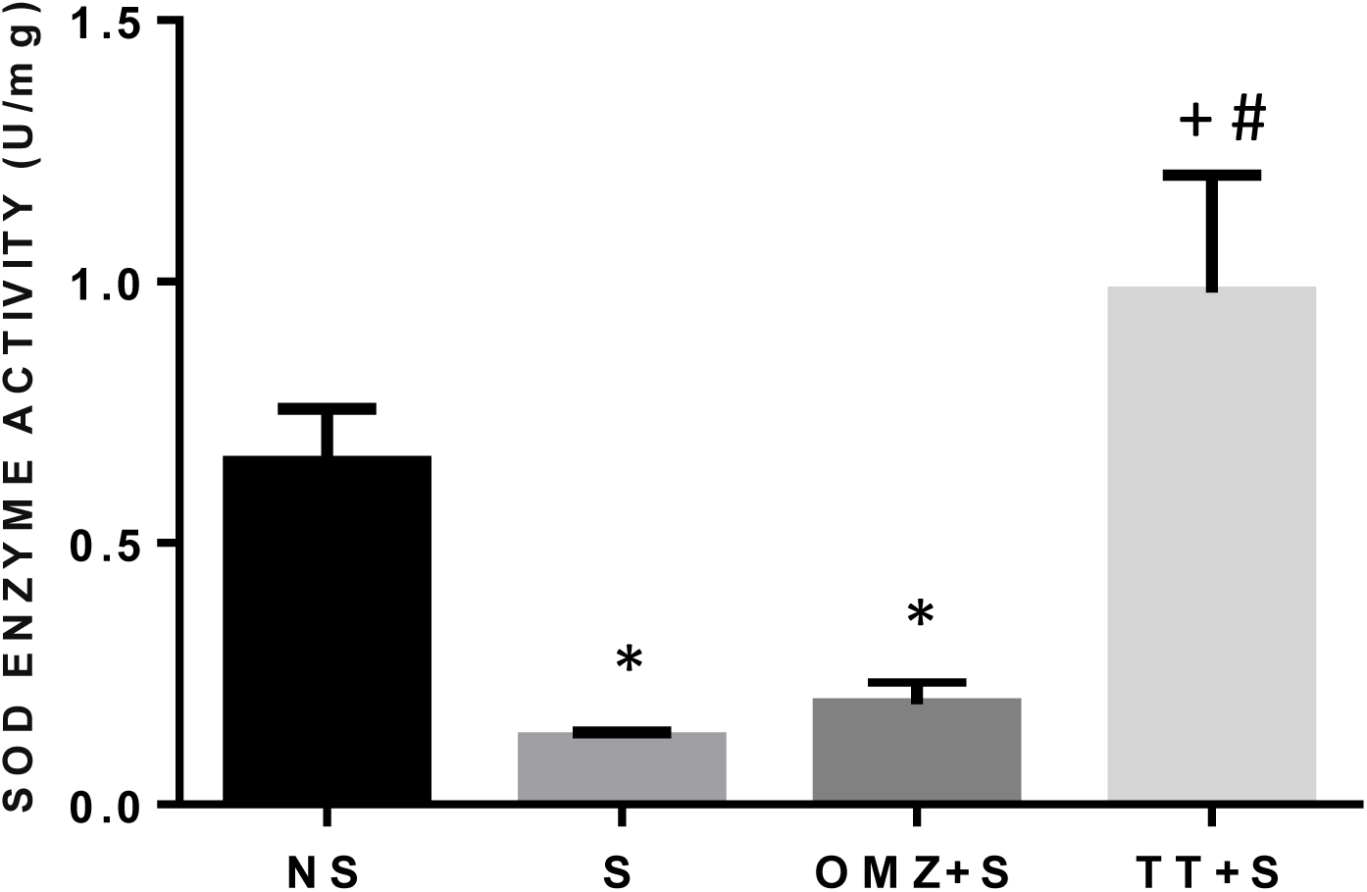
Effects of pre-treatment with tocotrienol (60 mg/kg BW) (TT) or omeprazole (20 mg/kg BW) (OMZ) on gastric SOD enzyme activity in rats exposed to water immersion restraint stress (n = 7). * vs non-stressed control (NS) (P < 0.05). ^+^ vs stressed control (S) (P < 0.05). ^#^ vs omeprazole (OMZ) (P<0.05).

The SOD mRNA expression was significantly lower in the intact mucosa as compared to rats exposed to stress (Fig 3). The gene expression of this enzyme however remains significantly low in rats pre-treated with either tocotrienol or omeprazole compared to the stressed control rats. The SOD activity was significantly reduced by 81% (P < 0.05) in the stressed group compared to the non-stressed control (Fig 4). Administration of tocotrienol leads to a significant (P = 0.0004) rise in SOD activity in rats exposed to stress. In comparison, pre-treatment with omeprazole produced a small but insignificant rise in SOD activity as compared to the stressed rats.

### Gastric Nitric Oxide (NO) and iNOS mRNA expression

**Fig 5:**
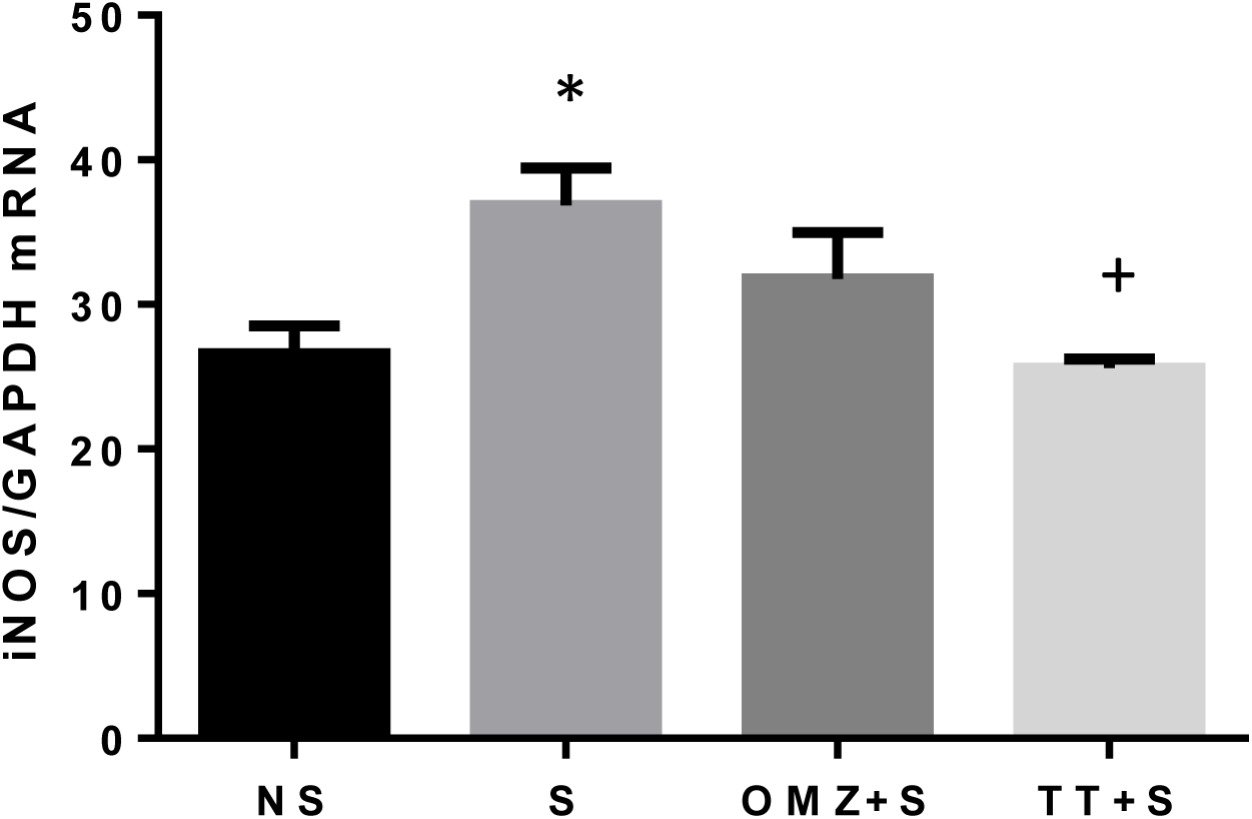
Effects of pre-treatment with tocotrienol (60 mg/kg BW) (TT) or omeprazole (20 mg/kg BW) (OMZ) on gastric inducible nitric oxide synthase (iNOS) mRNA expression in rats exposed to water immersion restraint stress (n = 7). * vs non-stressed control (NS) (P < 0.05).^+^vs stressed control (S) (P < 0.05).

**Fig 6:**
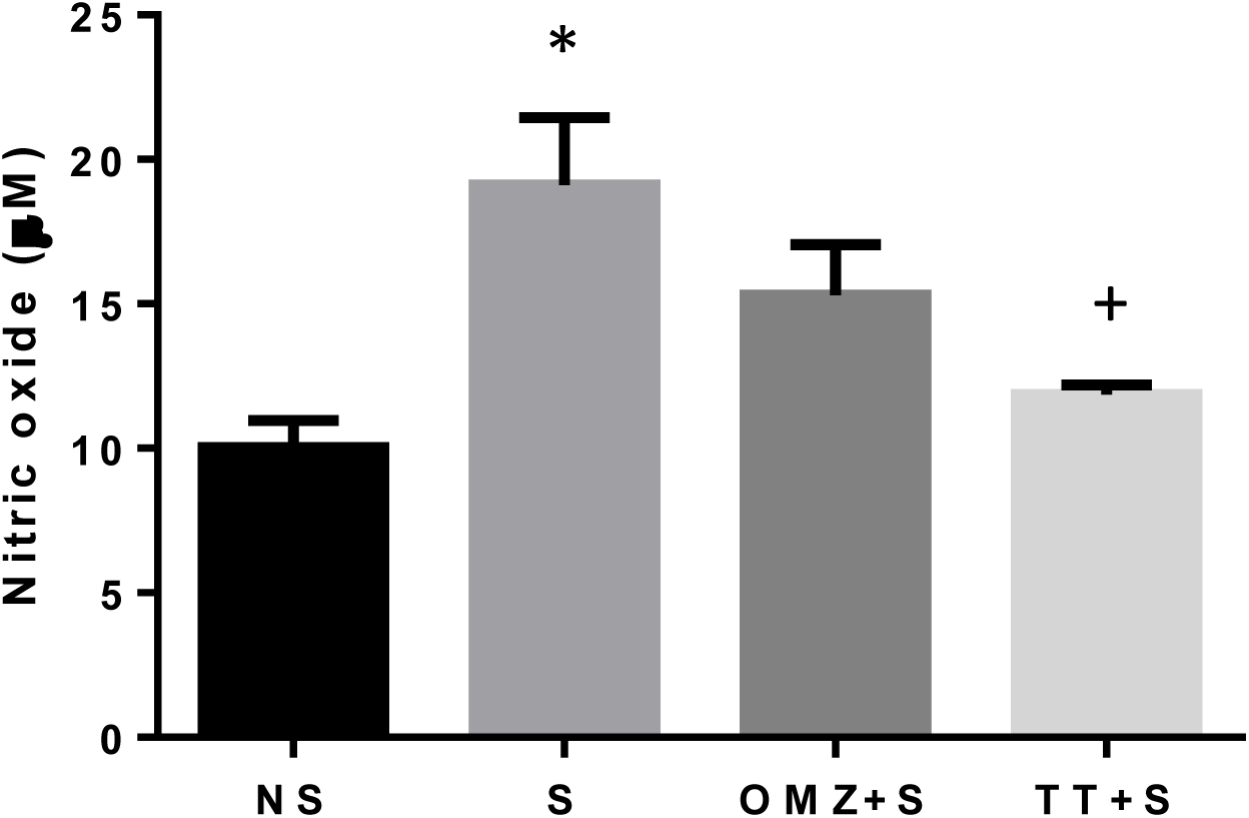
Effects of pre-treatment with tocotrienol (60 mg/kg BW) (TT) or omeprazole (20 mg/kg BW) (OMZ) on gastric nitric oxide (NO) concentration in rats exposed to water immersion restraint stress (n = 7). * vs non-stressed control (NS) (P < 0.05). ^+^ vs stressed control (S) (P < 0.05).

Exposure to 3.5 hours of stress resulted in 34% increase of iNOS gene expression in the gastric tissue as compared to the non-stressed control (Fig 5). This was accompanied by an increase in NO content in the stressed rats (Fig 6). However, tocotrienol pre-treatment significantly reduced this stress-induced elevation of both NO content (P = 0.0071) and iNOS gene expression (P = 0.0231). Omeprazole did not affect the expression of iNOS significantly. The same pattern was observed for gastric tissue NO content.

### Gastric TGF-α and IL-1β protein

**Fig 7:**
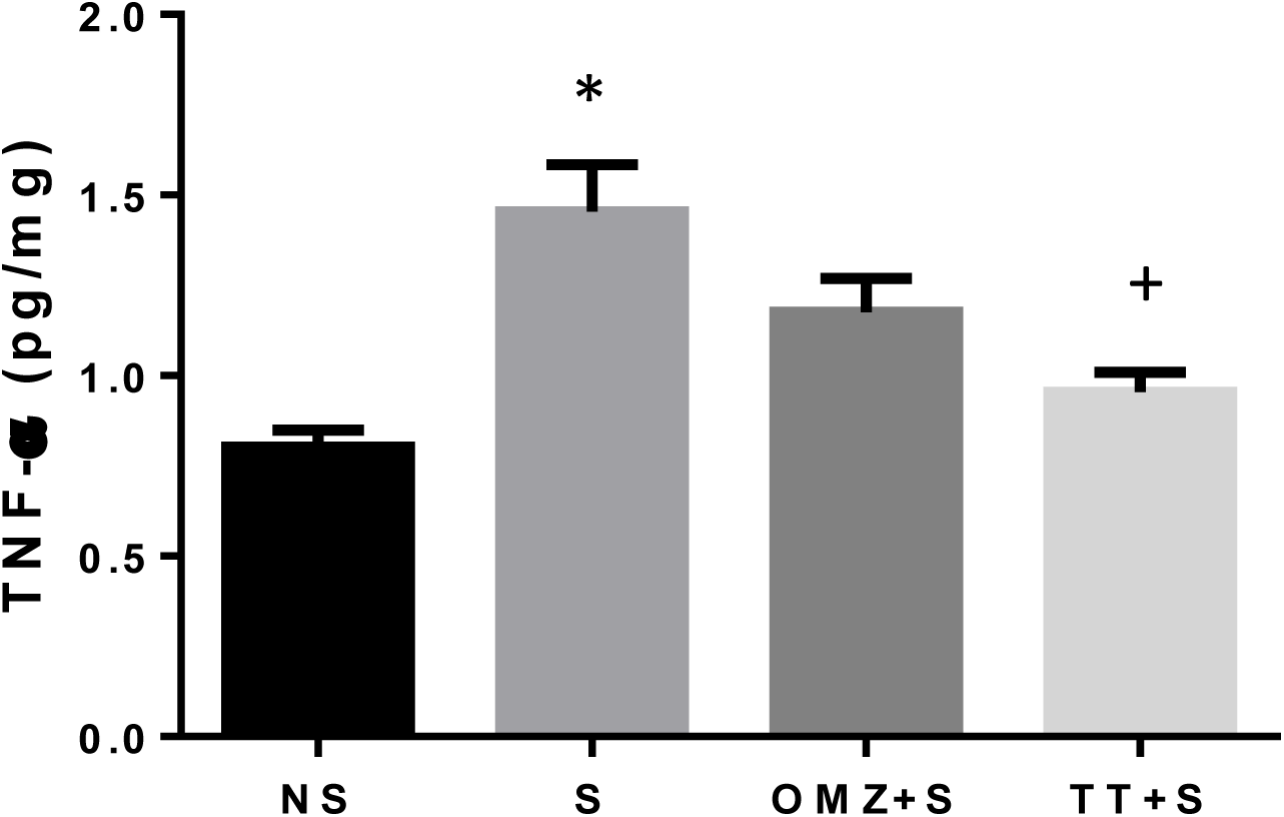
Effects of pre-treatment with tocotrienol (60 mg/kg BW) (TT) or omeprazole (20 mg/kg BW) (OMZ) on gastric Tumor necrosis factor alpha (TNF-α) protein concentration in rats exposed to water immersion restraint stress (n = 7). * vs non-stressed control (NS) (P < 0.05). ^+^ vs stressed control (S) (P < 0.05).

In intact control rats, the concentration of TNF-α in the gastric tissue remained at a low level, while rats exposed to stress had a two-fold higher content (P = 0.0001), as shown in Fig 7. Pre-treatment with tocotrienol was able to significantly (P = 0.0027) reduce the TNF-α compared to the stressed rats. Omeprazole pre-treatment, however, did not manage to restore the gastric tissue TNF-α level towards the non-stressed values.

**Fig 8:**
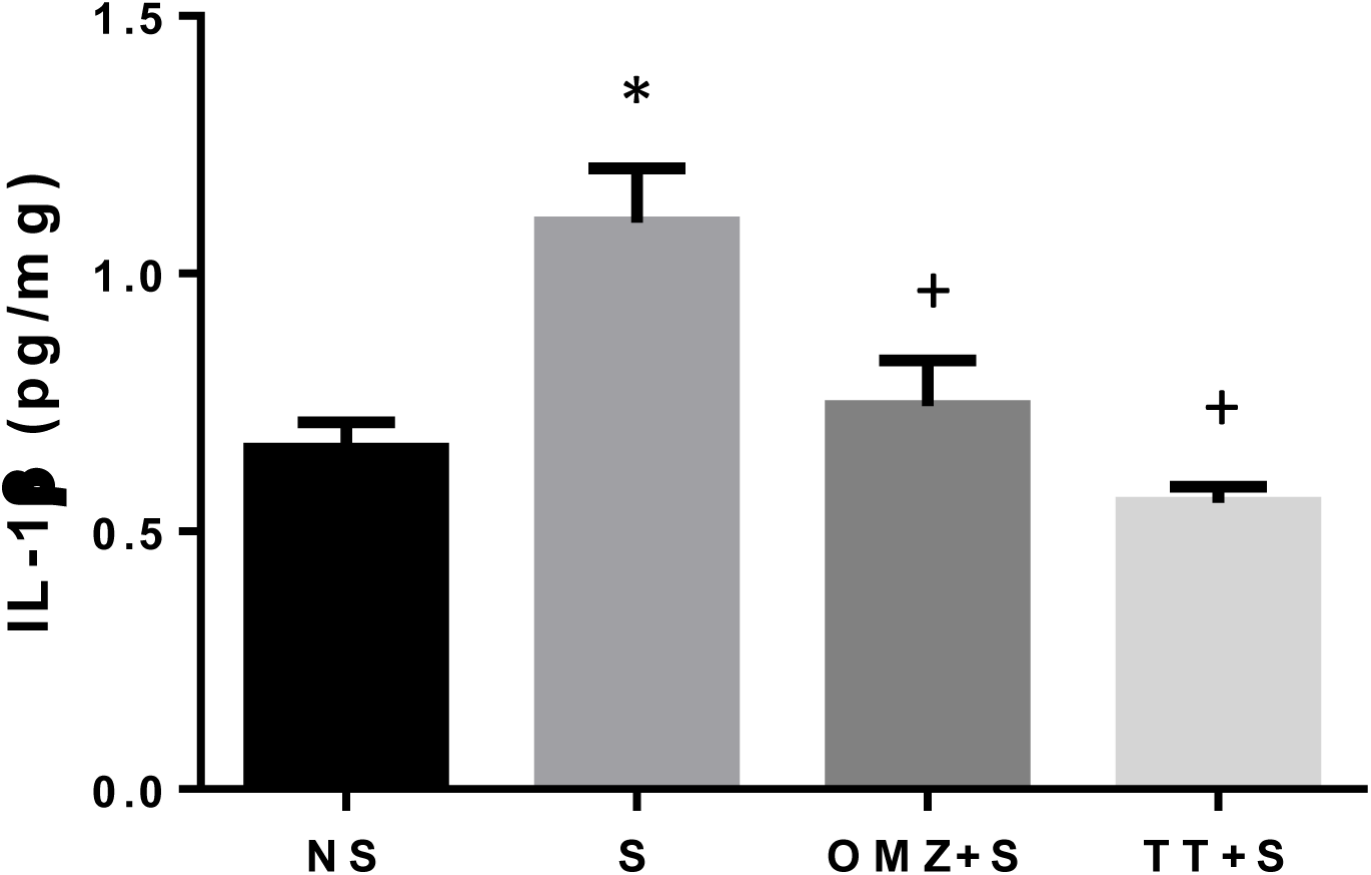
Effects of pre-treatment with tocotrienol (60 mg/kg BW) (TT) or omeprazole (20 mg/kg BW) (OMZ) on gastric Interleukin 1-beta (IL1-β) protein concentration in rats exposed to water immersion restraint stress (n = 7). * vs non-stressed control (NS) (P < 0.05). ^+^ vs stressed control (S) (P < 0.05).

Similarly, as shown in Fig 8, the gastric IL1-β in intact (non-stressed control) animals were significantly higher as compared to stressed control rats (P = 0.0020) after exposure to 3.5 hours of WIRS. Pre-treatment with tocotrienol (P = 0.0002) and omeprazole (P = 0.0127), prior to WIRS, led to a significant decrease in gastric IL1-β content, as compared to the stressed control. No differences between these groups or the non-stressed control were noted. Which suggest equal ability of tocotrienol and omeprazole in reducing this pro-inflammatory cytokine in rats exposed to stress.

## Discussion

Several studies have demonstrated the beneficial effect of vitamin E on ulcers or lesion formation in the stomach [7,22,24]. In the present study, gastric lesions developed in the glandular part of gastric mucosa in response to restraint plus water-immersion stress for 3.5 hours. The lesion was in the form of generalised erythema, haemorrhages and erosion of the mucosa. Research has shown the importance of maintaining a balance between the destructive and the protective capacity of the gastric mucosa to prevent formation of such mucosal injuries [2,4]. Supplementation with vitamin E such as tocopherol and tocotrienol have been shown to reduce the formation of stress-induced gastric lesions as reported in previous studies [24,25]. The findings observed in the study by Ibrahim et al. showed a significant reduction in mean gastric lesions in rats supplemented with palm-vitamin E (which contains 70% tocotrienol) and α-tocopherol (60 mg/kg body weight for 28 days). This is consistent with the findings in the present study, which have shown that tocotrienol was able to protect the gastric mucosa against stress-induced gastric injuries. Our findings demonstrate that pre-treatment of rats with either tocotrienol or omeprazole markedly reduced gastric mucosal damage induced by stress, which confirms the significant ability of these two agents in reducing stress-induced gastric injuries with stress exposure. However, the mechanisms whereby tocotrienol protects against stress-induced gastric mucosal injury remain unclear.

To better understand the mechanisms involved in the gastroprotective activity of tocotrienol and omeprazole, experimental models were developed in order to investigate the involvement of lipid peroxidation, antioxidant enzyme, cytokines, and NO in stress-induced gastric lesions. Nitric oxide is a double-edged weapon exerting either protective or destructive effects depending on the extent of NO synthesis [26]. It has been reported that NO generated from constitutive NOS (cNOS) plays an important role in gastric ulcer formation and healing [27], and considered to be beneficial in maintaining the mucosal integrity [28], whereas NO generated from iNOS participates in ulcer formation through the production of oxygen derived radical and their cytotoxic actions [29]. This suggests that NO production from iNOS may play a detrimental role in stress-induced gastric injuries. The present work showed a marked reduction in nitrite levels (an indicator of NO) in the tocotrienol-treated group, a change not observed in the omeprazole pre-treated group. These findings can be attributed to the ability of tocotrienol to downregulate the iNOS mRNA expression leading to decreased production of gastric mucosal NO. The nitrite levels is markedly elevated, which could be occurring due to stimulation of iNOS, which reacts with superoxide to form peroxynitrite, a potent cytotoxic oxidant causing gastric damage [30]. In addition, NO was reported to increase prostaglandin E_2_ (PGE_2_) biosynthesis *in vivo* through a CGMP independent mechanism, and it is possible to assume that NO might regulate the release and/ or the biosynthesis of PGE_2_ in the stomach after damage [31,32]. We had previously reported that there was an upregulation of cyclooxygenase-2 (COX-2) mRNA post stress which may be important in the production of PGE_2_ during the healing of ulcers in rats exposed to stress [7]. This could be explained by the increase in NO in stressed rats, which plays a role in PGE_2_ regulation. Konturek et al. [33] reported an increase in iNOS expression at the ulcer margins, which is a possible source of endogenous NO. They showed that ulcer healing can be interfered with by blocking the cyclooxygenase-prostaglandin system (COX-PG system) and inhibition of the NO system [33].

Moreover, COX-2, the inducible enzyme, is upregulated by pro-inflammatory cytokines and growth factors, where it mediates pathological reactions, such as inflammation [34]. Interestingly, we had previously shown an increase in COX-2 mRNA expression in rats with gastric mucosal lesions. Acute inflammation of gastric mucosa is known to be responsible for the enhanced permeability of blood vessels to activated neutrophils, resulting in an excessive infiltration of gastric mucosal tissue [11,35,36]. It was shown that the membrane of neutrophils exhibits NADPH oxidase activity, a key enzyme in the formation of superoxide radical anions [37,38]. We confirmed the overexpression of pro-inflammatory cytokines IL-1β and TNF-α which may play an essential role in the pathomechanism of WIRS-induced gastric damage, could explain the enhanced permeability of blood vessels to neutrophils, as has been previously reported [11,39].

Wilankar et al. [17] had previously described the activity of γ-tocotrienol as an anti-inflammatory agent, able to inhibit the pro-inflammatory cytokines. The tocotrienol mixture extracted from palm oil in this study contained a higher amount of γ-tocotrienol as compared to other isomers, which was almost 50%. This could contribute to its ability to reduce pro-inflammatory cytokines in this study. The effects of omeprazole on cytokines are quite different compared to tocotrienol; it was observed that IL1-β was reduced but the TNF-α concentration was increased compared to the non-stress control group. Further, IL-1β markedly increases infiltration of leucocytes in the superficial portion of scarred mucosa prior to ulcer recurrence [40], while enhancing ICAM-1 expression [41]. They found that sufficient inhibition of gastric acid by omeprazole inhibited both ulcer recurrence and responses, which suggested that acid may enhance gastric mucosal inflammation in response to IL1-β stimulation, resulting in gastric ulcers.

The enhanced expression and release of IL-1β and TNF-α could also contribute to the increased generation of reactive oxygen species in the gastric mucosa. Superoxide radical anions react with cellular membrane lipids, leading to the formation of lipid peroxides and giving rise to MDA. As shown in the present study, WIRS-induced gastric damage is associated with augmented reactive oxygen species (ROS)-induced lipid peroxidation manifested by an increase in MDA concentration. Gastric mucosa itself possesses an effective enzymatic system capable of scavenging ROS and preventing cell damage and injury to gastric mucosa [11,42]. Superoxide dismutase is the major antioxidative enzyme in gastric mucosa that catalyses the dismutation of the superoxide radical anion into a less noxious product, hydrogen peroxide (H_2_O_2_), which in turns undergoes further inactivation by the enzymatic activity of glutathione peroxidase (GPx). The increased in lipid peroxidation in this study was accompanied by an enhancement of antioxidative mechanisms, in particular, an increase in the mucosal expression of SOD, as shown in this study. This could be due to the depletion of the SOD enzymes with the presence of overwhelming radicals. However, treatment with tocotrienol reduces the lipid peroxidation, as shown by the reduction of MDA concentration. The SOD enzyme activity was also increased in rats pre-treated with tocotrienol, while the SOD gene expression remained low. The expression of these enzymes were high in the stressed control group but remained low in the tocotrienol and omeprazole groups, which did not differ when compared with rats not exposed to stress. It is possible that the depletion of the SOD enzyme due to excessive oxidative stress during stress exposure resulted in a normal physiological response by upregulating the SOD gene, where oxidative stress is known to cause the upregulation of antioxidant gene expression [43]. Thus, SOD enzyme expression remains low in animals treated with tocotrienol because there was no SOD depletion during stress possibly due to the supply of exogenous antioxidants, aka tocotrienol. However, a different outcome was observed by Hajiani et al. [44] where the administration of tocopherol in a large dose (100 mg/kg) for 6 weeks was able to increase the expression of SOD mRNA in the blood cells of rats. This contradictory finding suggests that tocopherol but not tocotrienol has a positive effect on SOD gene expression. It is, however, important not to rule out the possible effects of tocotrienol on SOD expression with a larger dose or a longer duration of the treatment period. This requires further research.

Enhanced lipid peroxidation and ROS production has been implicated in contributing to gastric mucosal lesion development in rats with WIRS [3,22,45]. It causes the degradation of the epithelial basement membrane components, completely altering the cell metabolism, and causing DNA damage [33]. In the present study, we observed a significant overexpression of SOD mRNA in the ulcerated mucosa, which occurs in response to the increase in inflammation and the increased expression of TNF-α mRNA. In parallel to the increased generation of ROS accompanying the induction of stress ulcers, that an agents with the ability to reduce inflammation improved ulcer healing by downregulation of SOD mRNA expression, which occurs in response to the inflammation in the area with ulcerations [33].

Although more famously known for its acid suppression activities, omeprazole, a proton pump inhibitor, possesses an antioxidative functions as well [46]. It has also been shown that omeprazole blocks stress-induced increased generation of hydroxyl radicals and associated lipid peroxidation and protein oxidation, indicating that its antioxidant role plays a major part in preventing oxidative damage [46]. They also reported that omeprazole prevents stress-induced DNA fragmentation, suggesting an anti-apoptotic role to block cell death during ulceration. Rats pre-treated with omeprazole had a lower lipid peroxidation level compared to the stressed control, but their SOD activity was not significantly different. These findings could be attributed to omeprazole’s action on hydroxyl radicals rather than the superoxide radicals, thus reducing the lipid peroxidation levels while not affecting SOD. The rationale for choosing to compare the effect of tocotrienol to omeprazole is due to the fact that it is currently one of the commonly prescribed drugs for treatment of peptic-ulcer disease in a clinical setting have also has antioxidant effect [46]. We had previously reported that omeprazole was able to reduce gastric acidity and increase the PGE_2_ level in response to stress [7].

## Conclusion

In summary, tocotrienol is effective in reducing stress-induced gastric damage, and these effects are partly supported by normalisation of gastric mucosal NO through reduction of iNOS expression and attenuation of pro-inflammatory cytokines IL-1β and TNF-α. It also exerts its antioxidant capabilities as shown by the reduction of lipid peroxidation and the increment of antioxidative enzymes SOD activity. In comparison to omeprazole, it exerts similar effectiveness in reducing stress-induce gastric lesions formation but with a more diverse mechanism of protection, particularly through its effect on gastric NO, TNF-α content, and SOD activity.
